# The Pros1/Tyro3 axis protects against periodontitis by modulating STAT/SOCS signalling

**DOI:** 10.1111/jcmm.14183

**Published:** 2019-02-07

**Authors:** Lei Jiang, Xiao Qing Chen, Ming Jing Gao, Wai Lee, Jie Zhou, Yun Fu Zhao, Guo Dong Wang

**Affiliations:** ^1^ Department of Stomatology, Changzheng Hospital Second Military Medical University Shanghai P. R. China

**Keywords:** periodontitis, Pros1, SOCS1/3, STAT1/3, Tyro3

## Abstract

Periodontitis, an oral inflammatory disease caused by periodontal pathogen infection, is the most prevalent chronic inflammatory disease and a major burden on healthcare. The TAM receptor tyrosine kinases (Tyro3, Axl and Mertk) and their ligands (Gas6 and Pros1) play a pivotal role in the resolution of inflammation and have been associated with chronic inflammatory and autoimmune diseases. In this study, we evaluated the effects of exogenous Pros1 in in vitro and in vivo models of periodontitis. We detected higher Pros1 but lower Tyro3 levels in inflamed gingival specimens of periodontitis patients compared with healthy controls. Moreover, Pros1 was mostly localized in the gingival epithelium of all specimens. In cultured human gingival epithelial cells (hGECs), *Porphyromonas gingivalis* LPS (*p.g*‐LPS) stimulation down‐regulated Pros1 and Tyro3. Exogenous Pros1 inhibited *p.g*‐LPS–induced production of TNF‐α, IL‐6, IL‐1β, MMP9/2 and RANKL in a Tyro3‐dependent manner as revealed by PCR, Western blot analysis, ELISA and gelatin zymography. Pros1 also restored Tyro3 expression down‐regulated by *p.g*‐LPS in hGECs. In rats treated with ligature and *p.g*‐LPS, administration of Pros1 attenuated periodontitis‐associated gingival inflammation and alveolar bone loss. Our mechanistic studies implicated SOCS1/3 and STAT1/3 as mediators of the in vitro and in vivo anti‐inflammatory effects of Pros1. Collectively, the findings from this work supported Pros1 as a novel anti‐inflammatory therapy for periodontitis.

## 1 INTRODUCTION

Periodontitis is a chronic inflammatory disease caused by periodontal pathogens such as *Porphyromonas gingivalis*, *Treponema denticola* and *Tannerella forsythia*.^1^ These pathogens release endotoxins which, in turn, elicit inflammatory responses in the periodontium, leading to degradation of gum tissues and alveolar bone resorption. Specifically, periodontal pathogen‐derived lipopolysaccharides (LPS) is reported to cause production of pro‐inflammatory factors, including tumour necrosis factor‐α (TNF‐α), interleukin (IL)‐1β, IL‐6 and matrix metalloproteinases (MMPs) by various resident cells of the periodontium through activation of Toll‐like receptor 2 (TLR2) or TLR4.^2^ The combined inflammatory responses stimulate osteoclastogenesis, resulting in alveolar bone loss.^3^ Periodontitis is the most prevalent chronic inflammatory disease and a major burden on healthcare systems. Severe periodontitis, which may result in tooth loss, affects 5%‐20% of most populations worldwide.^4^ In addition, periodontitis is an independent risk factor for several chronic diseases such as diabetes, cardiovascular disease and cancer.^5^, ^6^, ^7^ For instance, periodontitis is associated with increased risk for total cerebrovascular incidents, and in particular, non‐haemorrhagic stroke.^8^

The TAM receptor tyrosine kinases (RTKs) TYRO3, AXL and MERTK were identified as a distinct RTK subfamily in 1991.^9^, ^10^ Although TAM receptors have important functions in the adult nervous, reproductive and vascular systems, they are best known for their pivotal roles in the negative regulation of the immune system, functioning at the interface of innate and adaptive immunity. Together with their ligands GAS6 and Pros1 (also called protein S), they inhibit innate inflammatory response to pathogens by dendritic cells and macrophages, stimulate the phagocytic activity of antigen‐presenting cells and promote the maturation of natural killer cells.^11^ Not surprisingly, the TAM signalling is implicated in a number of chronic inflammatory and autoimmune diseases such as multiple sclerosis (MS)^12^ and systemic lupus erythematosus (SLE).^13^, ^14^ Mechanistically, the TAM receptors can associate with interferon (IFN)‐ receptor 1 (IFNAR1), and thereby, activate the suppressor of cytokine signalling proteins SOCS1 and SOCS3, subsequently inhibiting IFN‐I production.^15^ Accumulating evidence has suggested a role of IFN‐I in the development of periodontitis. Elevated levels of IFN‐ were detected in gingival tissues and plasma of periodontitis patients.^16^, ^17^ Moreover, the periodontitis‐associated pathogen *P gingivalis* or its LPS (*p.g*‐LPS) can stimulate IFN‐ production by macrophages through TLR signalling.^18^ Interestingly, loss of negative regulation on IFN‐I by TAM was reported to be responsible for the uncontrolled IFN‐1 production in a murine model of *P gingivalis*‐induced periodontitis,^19^ supporting a protective role of the TAM signalling against this oral inflammatory disease. However, the mechanisms underlying immunoregulation of TAM receptor tyrosine kinases or their ligands during periodontitis, especially for Pros1, was yet to be elucidated.

In this study, we evaluated the effects of exogenous Pros1 on *P gingivalis* LPS (*p.g*‐LPS)‐induced inflammation in human gingival epithelial cells (hGECs) in vitro as well as on ligature‐induced, *p.g*‐LPS–augmented periodontal inflammation and alveolar bone loss in vivo. Also, the mechanisms involving SOCS1/SOCS3 and STAT1/STAT3 were also investigated. The aim of the study was to clarify the specific role of Pros1/Tyro3 axis in regulating oral inflammatory disease such as periodontitis.

## 2 MATERIALS AND METHODS

### 2.1 Patients and tissue samples

Gingival specimens containing both epithelial and connective tissues were obtained during tooth extraction from 12 patients with chronic periodontitis and 8 healthy controls with non‐inflamed gingiva. All participants were 23‐62 years of age and had no history of smoking. This study was conducted in accordance with the Declaration of Helsinki and was approved by the Ethics Committee of Shanghai Chang Zheng Hospital (Shanghai, China). All study participants gave written informed consent. Gingival tissues were either fixed for histologic examinations or promptly frozen in liquid nitrogen and stored at −80°C until further use. The clinical features of the patients are presented in Table 1.

**Table 1:** Clinical data of periodontitis patients and control participants with non‐inflamed gingiva

	Control (n = 8)	Periodontitis (n = 12)	*P*‐value
Gender (M/F)	3/5	4/8	ns
Age (years)	38.2 ± 7	36.0 ± 9.7	ns
Body mass index (kg/m^2^)	24.3 ± 2.2	24.9 ± 2.6	ns
Periodontal data			
PD (mm)	1.7 ± 0.3	4.8 ± 0.4	<0.01
CAL (mm)	1.5 ± 0.7	3.9 ± 0.6	<0.01
GI	0.8 ± 0.3	2.6 ± 0.2	<0.01
PLI	0.5 ± 0.1	1.7 ± 0.6	<0.01
SBI	1.1 ± 0.3	2.9 ± 0.8	<0.01

Data represent the mean ± SD. PD, periodontal probing depth; CAL, clinical attachment level; GI, gingival index; PLI, plaque index; SBI, sulcus bleeding index.

### 2.2 Cell culture, reagents and antibodies

Primary hGECs were obtained from CELLnTEC (Stauffacherstrasse, Switzerland) and maintained in CnT‐PR media (CELLnTEC) supplemented with 100 U/mL penicillin and 100 mg/mL streptomycin at 37°C, 5% CO_2 _in a humidified incubator. *p.g*‐LPS was obtained from InvivoGen (San Diego, CA, USA). Human Pros1 protein was from Enzyme Research Laboratories (South Bend, IN, USA). Anti‐TYRO3 antibody was from R&D Biosystems (Minneapolis, MN, USA).

### 2.3 Animals

Male Sprague‐Dawley rats weighting 250‐300 g were purchased from the Chinese Academy of Sciences (Shanghai, China). Rats were randomly divided into five groups (n = 6 per group): control (no‐treatment), ligature (ligature only), ligature + *p.g*‐LPS, +PBS (ligature + *p.g*‐LPS + PBS) and +Pros1 (ligature + *p.g*‐LPS + Pros1). The rats were anaesthetized with an intraperitoneal injection of Zoletil (0.4 mL/kg, Virbac Laboratories, Carros, France) and Rompun (10 mg/kg, Bayer Korea Ltd., Seoul, South Korea). An elastic ligature was placed between the first and second right maxillary molars to induce periodontitis. The ligature + *p.g*‐LPS, +PBS and +Pros1 groups received 20 L of 1 mg/mL *p.g*‐LPS three times a week for 2 weeks. The toxin was injected into the palatal gingivae around the first and second maxillary molars. In addition, the +Pros1 group received daily subcutaneous injection of 20 μg Pros1 in 30 μL PBS, whereas the +PBS group received PBS solution only. All rats were killed at the end of treatment. All animal studies were approved by the Animal Care and Use Committee at the Second Military Medical University.

### 2.4 Quantitative real‐time PCR

Total RNA was isolated using TRIzol reagent (Invitrogen) according to manufacturer's instructions and reverse‐transcribed into complementary DNA (cDNA). Quantitative PCR was carried out on a LightCycler system (Roche LifeScience) with SYBR Green master mix (Roche Applied Science). Primer sequences used in the PCR reactions are listed in Table 2. Data were normalized to that of β‐actin in the same reaction. Relative expression was calculated using the 2^−∆∆Ct^ method.

**Table 2:** Sequences of primers used in qRT‐PCR

Gene	GenBank No.	Primer sequence (5′‐3′)
*Homo sapiens* (human)		
Pros1	NM_000313	F: GGCGTGATACTGTACGCAGA; R: TCCGGCTTAAAAAGGGGTCC
Tyro3	NM_001330264	F: CAAACTGCCTGTCAAGTGGC; R: TGAGATCATACACGTCCTCCA
Gas6	NM_000820	F: CATCAACCATGGCATGTGGC; R: TTCTCCGTTCAGCCAGTTCC
Axl	NM_001278599	F: CACCCCAGAGGTGCTAATGG; R: GGTGGACTGGCTGTGCTT
Mertk	NM_006343	F: GCCCCATCAGTAGCACCTTT; R: TGCACGTAGCATTGTGGACT
TNF‐α	NM_000594	F: CATCTTCTCGAACCCCGAGT; R: ATGAGGTACAGGCCCTCTGAT
IL‐6	NM_000600	F: CAGCCCTGAGAAAGGAGACAT; R: TTGCATCTAGATTCTTTGCCTTTTT
IL‐1β	NM_000576	F: CTGAGCTCGCCAGTGAAATG; R: CATGGCCACAACAACTGACG
MMP‐9	NM_004994	F: CCTGGGCAGATTCCAAACCT; R: GTACACGCGAGTGAAGGTGA
MMP‐2	NM_001127891	F: TGATGGCATCGCTCAGATCC; R: GGCCTCGTATACCGCATCAA
RANKL	NM_003701	F: CCAGCAGAGACTACACCAAGT; R: TAGGATCCATCTGCGCTCTG
*Rattus norvegicus* (Norway rat)		
Pros1	XM_008765045	F: AAGGGCTCCTACTACCCTGG; R: GCCAGAATCCACCAAGGACA
Tyro3	NM_017092	F: GTGGAAGGAACTACGGCCAA; R: GATGTACGGCTGTGAGGAGG
TNF‐α	NM_012675	F: GTCGTAGCAAACCACCAAGC; R: TCCCTCAGGGGTGTCCTTAG
IL‐6	NM_012589	F: ACAAGTCCGGAGAGGAGACT; R: ACAGTGCATCATCGCTGTTC
MMP‐9	NM_031055	F: CGGCAAACCCTGCGTATTTC; R: GTTGCCCCCAGTTACAGTGA
MMP‐2	NM_031054	F: TTGCTCAGATCCGTGGTGAG; R: GGTCAGTGGCTTGGGGTATC
RANKL	NM_057149	F: CATGAAACCTCAGGGAGCGT; R: GTTGGACACCTGGACGCTAA

### 2.5 Western blot analysis

Cells were lysed using RIPA lysis buffer containing protein kinase and phosphatase inhibitors for 30 minutes on ice. Tissue samples were homogenized by sonication and proteins were extracted. Protein concentrations were determined using a BCA kit (Thermo Fisher). Samples (20 μg) were separated by SDS‐polyacrylamide gel electrophoresis and transferred to polyvinylidene difluoride (PVDF) membranes. After blocked in 5% skim milk for 1 hour, the membranes were probed with primary antibodies towards RANKL (SC‐7627, Santa Cruz Biotechnology), Pros1 (SC‐271326, Santa Cruz Biotechnology), Tyro3 (SC‐271326, Santa Cruz Biotechnology), SOCS1 (ab9870, Abcam), SOCS3 (ab16030, Abcam), STAT1 (ab31369, Abcam), phospho (p)‐STAT1 (phosphor Y701, ab30645, Abcam), STAT3 (cat. no. 12640, Cell Signaling) and p‐STAT3 (Tyr705, cat. no. 07‐2173, Millipore), respectively, at 4°C overnight. The membranes were subsequently incubated with a horseradish peroxidase (HRP)‐conjugated secondary antibody at room temperature for 2 hours, and the protein bands were visualized using enhanced chemiluminescence. All data were normalized to ‐actin.

### 2.6 Enzyme‐linked immunosorbent assay

Concentrations of the inflammatory cytokines TNFα, IL‐6 and IL‐1β in hGEC culture supernatants were determined using Quantikine enzyme‐linked immunosorbent assay (ELISA) kits (R&D Systems, Minneapolis, MN, USA) following manufacturer's instructions. Data were normalized to cell number in each test.

### 2.7 Gelatin zymography

The enzymatic activities of MMP‐2 and MMP‐9 in hGEC culture media were determined using a gelatin zymography system (Novex Life Technology, Carlsbad, CA, USA). In brief, proteins in the medium were separated under non‐reducing denaturing conditions on a 10% SDS‐polyacrylamide gel containing 1 mg/mL gelatine. After washing with 2.5% Triton X‐100 and overnight incubation at 37C, the gels were stained with 0.1% Coomassie blue R‐250 for 4 hours and immersed into a buffer containing 45% methanol and 10% acetic acid. Gel images were obtained on a Transilluminator (Diagnostic Instruments, Sterling Heights, MI, USA).

### 2.8 Micro‐CT analysis

Micro‐CT imaging was performed 2 weeks after periodontitis induction on a SkyScan microCT scanner (Bruker microCT, Kontich, Belgium). The maxillary jaws were hemisected and the right half of the block samples were cut into 18‐μm slices and fixed in 4.0% paraformaldehyde. Computed tomography was conducted with a voltage of 50 kV and an electrical current of 455 μA. Three‐dimensional images were acquired using the Bruker microCT version 1.1 software. The distance from the cement‐enamel junction (CEJ) to the alveolar bone crest (ABC) at the palatal groove site of M2 was measured as previously described.^20^

### 2.9 Histology and immunohistochemistry

The maxilla specimens were fixed in 4% paraformaldehyde for at least 24 hours and decalcified in 10% EDTA solution for 6 weeks at 4°C, with the solution exchanged every other day. The decalcified specimens were dehydrated, embedded in paraffin and cut into 4‐μm sections. After dewaxing and rehydration, the sections were stained with haematoxylin and eosin (HE) for histological analysis. Expression and distribution of Pros1, Tyro3 and RANKL were detected by immunohistochemical staining.

### 2.10 TRAP staining

The maxilla sections were subjected to tartrate‐resistant acid phosphatase (TRAP) staining using a leukocyte acid phosphatase kit (Sigma‐Aldrich) following manufacturer's instructions. The specimens were counterstained with haematoxylin. TRAP‐positive multinucleated cells (active osteoclasts) on the surface of alveolar bone around the first molar were counted. The results were expressed as the total cell count from four different visual fields of each section.

### 2.11 Statistical analysis

All results are presented as mean ± SD. Data were analysed using SPSS 22.0 software (SPSS Inc, Chicago, IL, USA). Each experiment was performed in triplicate. Data from different groups were compared using one‐way ANOVA with the Hochberg test or two‐sample *t* test. A *P *< 0.05 was considered statistically significant.

## 3 RESULTS

### 3.1 Pros1 is up‐regulated in gingival tissues of chronic periodontitis patients

**Figure 1:**
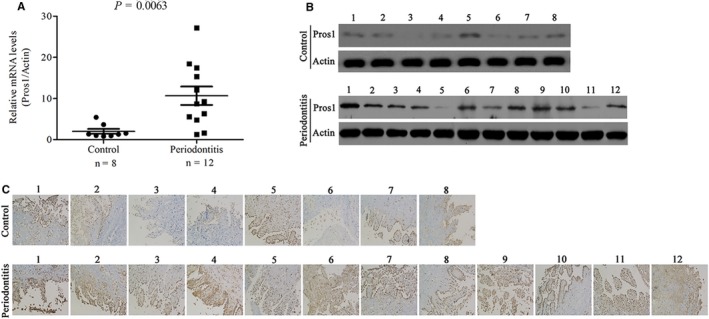
Pros1 is up‐regulated in gingival tissues of chronic periodontitis patients. (A‐C) Pros1 expression in gingival tissues from periodontitis patients (periodontitis, n = 12) and healthy controls (Control, n = 8) by qRT‐PCR (A, *P = *0.0063), Western blot analysis (B) and immunohistochemical staining (C)

The TAM receptors Tyro3, Axl and Mertk and their ligands Gas6 and Pros1 were detected by qRT‐PCR in gingival tissues of patients with chronic periodontitis as well as healthy controls with non‐inflamed gingiva. While Gas6, Axl and Mertk were detected at similar levels in periodontitis and control gingiva (Figure S1A, C and D), Pros1 was found to be up‐regulated and Tyro3 down‐regulated in periodontitis specimens compared with controls (Figure 1A, Figure S1B). Western blot analysis and immunohistochemical staining confirmed increased Pros1 protein expression in the inflamed gingiva compared with non‐inflamed control (Figure 1B and C). Furthermore, positive Pros1 staining was mainly detected in the gingival epithelium of both periodontitis patients and non‐inflamed controls (Figure 1C).

### 3.2 Pros1 inhibits *p.g*‐LPS–induced inflammation in hGECs via Tyro3

**Figure 2:**
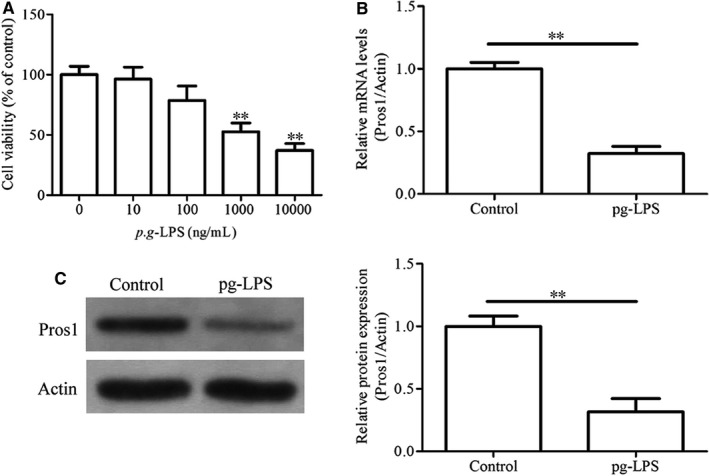
*p.g‐*LPS down‐regulates Pros1 in hGECs. (A) hGECs were treated with *p.g*‐LPS at indicated concentrations for 24 hours. Cell viability was determined by the MTT assay. Untreated cells (0 ng/mL *p.g*‐LPS) were used as control. n = 3, **P < 0.01 vs untreated cells. (B, C) hGECs were treated with 10 g/mL *p.g*‐LPS for 24 hours. Pros1 mRNA and protein levels were determined by qRT‐PCR (B) and Western blot analysis (C) respectively. n = 3, **P < 0.01

**Figure 3:**
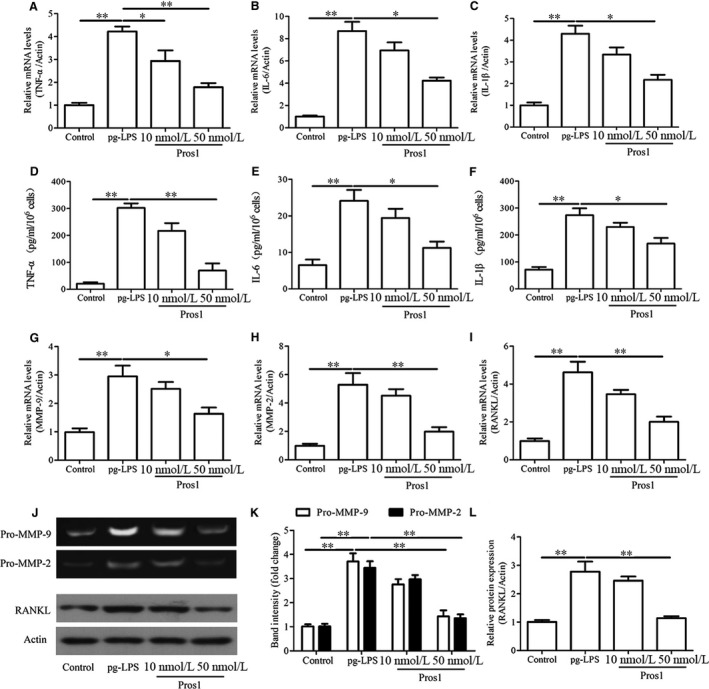
Pros1 inhibits *p.g*‐LPS–induced inflammation in hGECs. hGECs were treated with *p.g*‐LPS (1 μg/mL) and Pros1 (10 or 50 nmol/L), alone or in combination as indicated for 24 hours. Untreated cells (Control) served as control. (A‐C, G‐I) TNF‐α (A), IL‐6 (B), IL‐1β (C), MMP‐9 (G), MMP‐2 (H) and RANKL (I) mRNA levels by qRT‐PCR. (D‐F) TNF‐α (D), IL‐6 (E) and IL‐1β (F) protein concentrations in the culture supernatants by ELISA. (J, K) The enzymatic activities of MMP‐9 and MMP‐2 in the culture supernatants by gelatin zymography. (J, L) RANKL protein levels by Western blot analysis. n = 3, *P < 0.05, **P < 0.01

**Figure 4:**
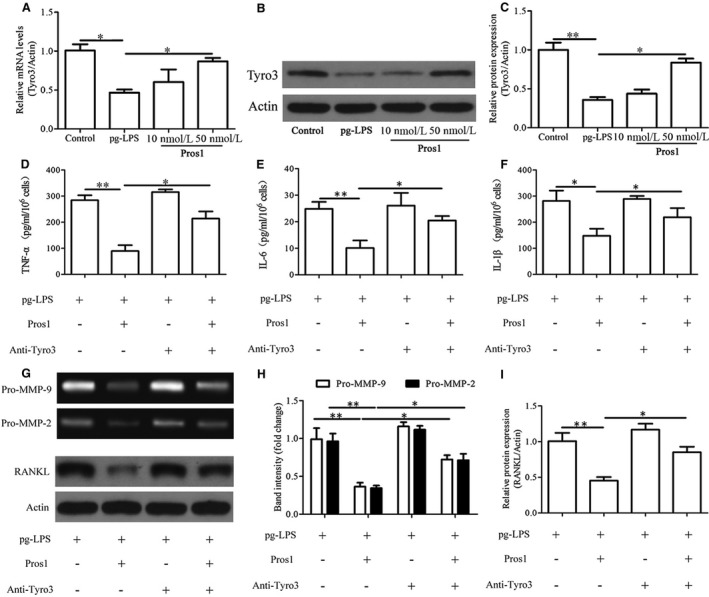
Pros1 inhibits *p.g*‐LPS–induced inflammation in hGECs via Tyro3. (A‐C) hGECs were treated with *p.g*‐LPS (1 μg/mL) and Pros1 (10 or 50 nmol/L), alone or in combination as indicated for 24 hours. Untreated cells (Control) served as control. Tyro3 mRNA (A) and protein (B, C) levels were determined by qRT‐PCR and Western blot analysis respectively. (D‐I) hGECs were treated with *p.g*‐LPS (1 μg/mL), Pros1 (50 nmol/L) and anti‐Tyro3 antibody (10 μg/mL), alone or in combination as indicated for 24 hours. (D‐F) TNF‐α (D), IL‐6 (E) and IL‐1β (F) protein concentrations in the culture supernatants were determined by ELISA. (G, H) The enzymatic activities of MMP‐9 and MMP‐2 in the culture supernatants were assessed by gelatin zymography. (G, I) RANKL protein levels were determined by Western blot analysis. n = 3, *P < 0.05, **P < 0.01

Stimulation of hGECs with increasing concentrations of *p.g*‐LPS for 24 hours led to dose‐dependent decreases in cell viability as indicated by the MTT assay (Figure 2A). The decreased cell viability of *p.g*‐LPS–treated cells was accompanied by significantly reduced Pros1 mRNA and protein expression as assessed by qRT‐PCR and Western blot analysis respectively (Figure 2B and C). In addition, stimulation with *p.g*‐LPS (1 g/mL) for 24 hours markedly increased the expression and secretion of the inflammatory cytokines TNF‐α, IL‐6 and IL‐1β as revealed by qRT‐PCR and ELISA tests (Figure 3A‐F). *p.g*‐LPS also up‐regulated the mRNA and protein expression of RANKL (Figure 3I, J and L), a key factor in osteoclastogenesis and bone resorption in periodontitis.^21^ Moreover, *p.g*‐LPS increased the mRNA expression and enzymatic activities of MMP‐9/2 as indicated by qRT‐PCR (Figure 3G and H) and gelatin zymography tests (Figure 3J and K) respectively. The productions of these pro‐inflammatory factors induced by *p.g*‐LPS were effectively attenuated by treatment with Pros1 (10 nmol/L and/or 50 nmol/L) (Figure 3A‐L). To examine the mechanisms involved in the anti‐inflammatory effects of Pros1, we evaluated the TAM receptor Tyro3. Stimulation with *p.g*‐LPS (1 g/mL) for 24 h significant down‐regulated Tyro3 mRNA and protein expression in hGECs, and treatment with Pros1 (50 nmol/L) restored Tyro3 expression down‐regulated by *p.g*‐LPS (Figure 4A‐C). Blocking Tyro3 signalling by a neutralizing antibody towards Tyro3 restored *p.g*‐LPS–induced production of TNF‐α, IL‐6, IL‐1β, MMP9/2 and RANKL inhibited by Pros1 (Figure 4D‐I). Collectively, these data supported that Pros1 signals through Tyro3 to inhibit *p.g*‐LPS–induced inflammation in hGECs.

### 3.3 The Pros1/Tyro3 signalling ameliorates *p.g*‐LPS–induced inflammation in hGECs via SOCS1/3 and STAT1/3

**Figure 5:**
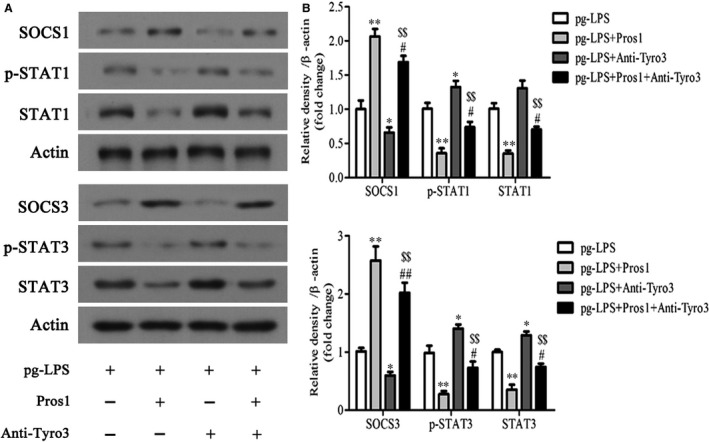
The Pros1/Tyro3 signalling ameliorates *p.g*‐LPS–induced inflammation in hGECs via SOCS1/3 and STAT1/3. hGECs were treated with *p.g*‐LPS (1 μg/mL), Pros1 (50 nmol/L) and anti‐Tyro3 antibody (10 μg/mL), alone or in combination as indicated for 24 hours. (A‐C) SOCS1, p‐STAT1, STAT1, SOCS3, p‐STAT3 and STAT3 protein levels were determined by Western blot analysis. Representative gel images (A) and relative protein expression by densitometric analysis (B, C) are shown. n = 3; *P < 0.05, **P < 0.01 vs *p.g*‐LPS; *P* < 0.05, *P* < 0.01 vs *p.g*‐LPS +Pros1; ^$$^ *P* < 0.01 vs *p.g*‐LPS + anti‐Tyro3

To unveil molecular pathways acting downstream of Pros1/Tyro3, we assessed SOCS1/3 and STAT1/3 by Western blot analysis. The results showed increased SOCS1/3 expression along with decreased STAT1/3 expression and phosphorylation in *p.g*‐LPS–stimulated hGECs in response to Pros1 treatment (Figure 5A‐C). Moreover, treatment with anti‐Tyro3 antibody reversed changes in SOCS1/3 and STAT1/3 induced by Pros1 (Figure 5A‐C). These data supported SOCS1/3 and STAT1/3 as mediators of the anti‐inflammatory effects of Pros1 acting downstream of Tyro3.

### 3.4 Pros1 reduces osteoclastogenesis and alveolar bone loss in periodontitis rats

**Figure 6:**
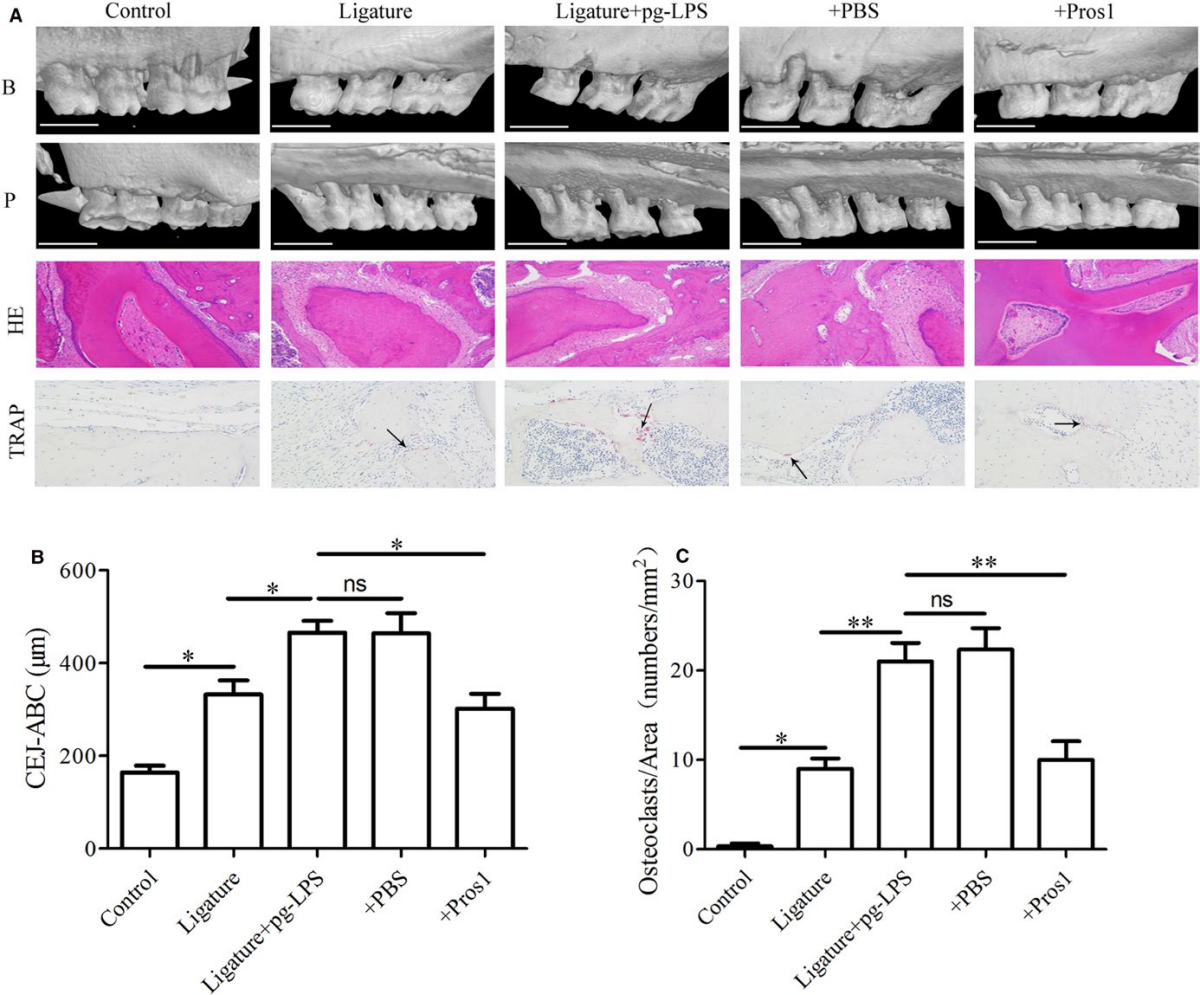
Pros1 reduces alveolar bone loss and osteoclastogenesis in periodontitis rats. Five groups of rats (n = 6 per group): Control (no‐treatment), Ligature (ligature only), Ligature + *p.g*‐LPS, Ligature + *p.g*‐LPS + PBS and Ligature + *p.g*‐LPS + Pros1 were treated as described in Section 2 for 2 weeks. (A) Representative images of micro‐CT, HE staining and TRAP staining of the maxilla sections. Arrows indicate TRAP‐positive multinucleated cells (active osteoclasts) between maxillary first and second molar and second and third molar. B, buccal surface; P, palatal surface; scale bars = 1 mm (micro‐CT); magnification = 200 × (HE and TRAP staining). (B) Bone levels evaluated by the average distance from cemento‐enamel junction (CEJ) to the alveolar bone crest (ABC) at the palatal groove site. n = 6, *P < 0.05, ns = nonspecific. (C) Periodontal osteoclastogenesis evaluated by the density of TRAP‐positive multinucleated cells on the surface of alveolar bone around the first molar. n = 6, *P < 0.05, **P < 0.01, ns = non‐specific

We subsequently examined the effects of Pros1 in rats subjected to combinatory treatment with ligature and *p.g*‐LPS. Micro‐CT images of the buccal and palatal surface and HE staining of maxilla sections revealed a normal periodontium structure with clearly defined gingiva, periodontal ligament, alveolar bone and cementum in the sham group (Figure 6A). At 2 weeks after periodontitis induction, the ligature and ligature + *p.g*‐LPS groups showed significant alveolar bone loss compared with sham as indicated by greater distances between the cemento‐enamel junction (CEJ) and the alveolar bone crest (ABC) at the palatal groove site of M2 (Figure 6A and B). Compared with sham, the ligature and ligature + *p.g*‐LPS groups also exhibited greater positive TRAP staining of the gingiva and higher numbers of TRAP‐positive multinucleated cells (active osteoclasts) on the surface of alveolar bone around the first molar (Figure 6A and C), indicating increased osteoclastogenesis in these two groups. The alveolar bone loss and osteoclastogenesis were more severe in the ligature + *p.g*‐LPS group compared with ligature only (Figure 6A‐C). Daily subcutaneous injection of 20 μg Pros1 in the ligature + *p.g*‐LPS group attenuated periodontitis‐mediated alveolar bone loss and osteoclastogenesis, showing a CEJ‐ABC distance and osteoclast number similar to those of the ligature only group (Figure 6B and C). These data supported the protective role of Pros1 against periodontitis‐associated structural damage in vivo.

### 3.5 Pros1 attenuates periodontal inflammation in periodontitis rats

**Figure 7:**
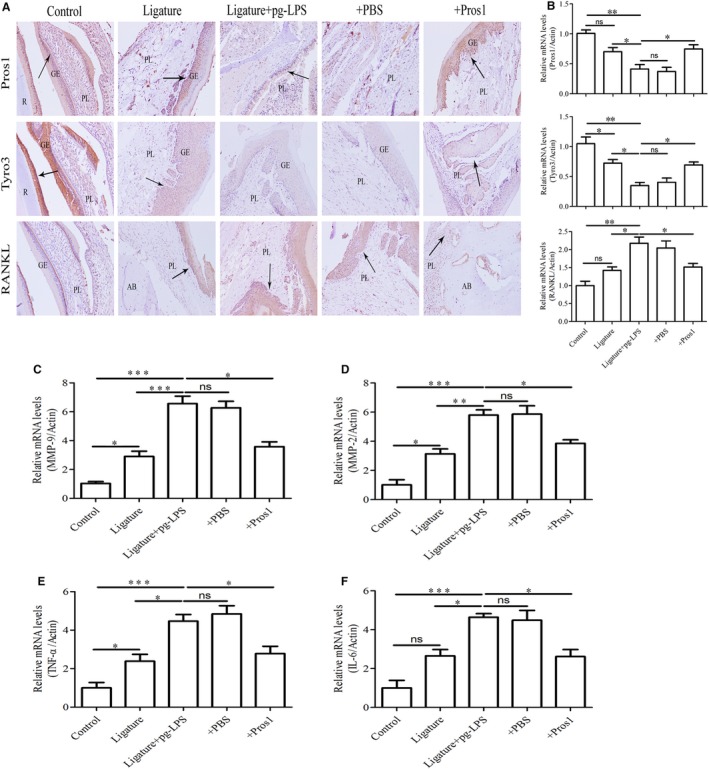
Pros1 attenuates periodontal inflammation in periodontitis rats. Five groups of rats (n = 6 per group): Control (no‐treatment), Ligature (ligature only), Ligature + *p.g*‐LPS, Ligature + *p.g*‐LPS + PBS and Ligature + *p.g*‐LPS + Pros1 were treated as described in Section 2 for 2 weeks. (A) Representative images of immunohistochemical staining of the maxilla sections for Pros1, Tyro3 and RANKL. GE, gingival epithelium; PL, periodontal ligament; AB, alveolar bone; R, root. (B) Pros1, Tyro3 and RANKL mRNA levels in the periodontium by qRT‐PCR. (C‐F) MMP‐9 (C), MMP‐2 (D), TNF‐α (E) and IL‐6 (F) mRNA levels in the periodontium by qRT‐PCR. n = 6, *P < 0.05, **P < 0.01, ***P < 0.001, ns = non‐specific

We next assessed Pros1, Tyro3 and RNAKL levels in periodontal tissues of periodontitis and sham rats. Immunohistochemical staining and qRT‐PCR analysis revealed lower Pros1 and Tyro3 and higher RANKL expression in the ligature + *p.g*‐LPS group than the sham or the ligature only group (Figure 7A and B). In all treatment groups, RANKL‐positive cells were mainly detected surrounding the alveolar bone surface, and Pros1 and Tyro3 signals were mostly localized in gingival epithelial cells (Figure 7A). The ligature + *p.g*‐LPS group also exhibited higher periodontal MMP‐9, MMP‐2, TNF‐ and IL‐6 mRNA expression compared with sham or ligature only (Figure 7C‐F). Treatment of the ligature + *p.g*‐LPS group with Pros1 increased Pros1 and Tyro3 expression and reduced RANKL, MMP‐9, MMP‐2, TNF‐ and IL‐6 expression, showing levels similar to those of the ligature only group (Figure 7A‐F). These data supported that Pros1 protects against structural damages in periodontitis rats by ameliorating periodontitis‐associated inflammation and osteoclastogenesis.

### 3.6 Pros1 protects against periodontitis via SOCS1/3 and STAT1/3

**Figure 8:**
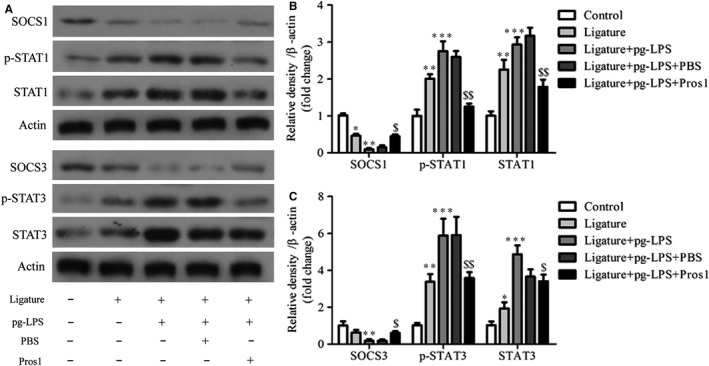
Pros1 protects against periodontitis via SOCS1/3 and STAT1/3. Five groups of rats (n = 6 per group): Control (no‐treatment), Ligature (ligature only), Ligature + *p.g*‐LPS, Ligature + *p.g*‐LPS + PBS and Ligature + *p.g*‐LPS + Pros1 were treated as described in Section 2 for 2 weeks. SOCS1, p‐STAT1, STAT1, SOCS3, p‐STAT3 and STAT3 protein levels in the periodontium were determined by Western blot analysis. Representative gel images (A) and relative protein expression by densitometric analysis (B and C) are shown. n = 6; *P < 0.05, **P < 0.01, ***P < 0.001 vs Control; ^$^ *P* < 0.05, ^$$^ *P* < 0.01 vs Ligature + *p.g*‐LPS + PBS

To investigate the mechanisms involved in the protective effects of Pros1 against periodontitis in rats, we evaluated rat periodontal SOCS1/3 and STAT1/3 by Western blot analysis. We found that ligature treatment led to decreased SOCS1/3 expression along with increased STAT1/3 expression and phosphorylation in the periodontium, and these ligature‐induced changes were augmented by administration of *p.g*‐LPS (Figure 8A‐C). Treatment of the ligature + *p.g*‐LPS rats with Pros1 increased SOCS1/3 expression and inhibited STAT1/3 expression and phosphorylation (Figure 8A‐C). These data supported SOCS1/3 and STAT1/3 as mediators of the protective effects of Pros1 against periodontitis in rats.

## 4 DISCUSSION

Pros1 primarily functions as a natural anticoagulant by controlling thrombin generation and fibrinolysis. As a cofactor to the activated protein C (APC) in the degradation of activated factors V (FVa) and VIII (FVIIIa), Pros1 negatively regulates prothrombinase and tenase activities in the coagulation cascade.^22^, ^23^ PS also has an APC‐independent anticoagulant activity by acting as a cofactor of tissue factor pathway inhibitor (TFPI), stimulating the inhibition of factor Xa.^24^, ^25^ Thus, it is not unexpected that partial Pros1 deficiency is associated with enhanced risk of venous thromboembolism^26^ while the carriers of a double mutated gene suffer from purpura fulminans that demands fresh plasma administration.^27^

Circulating Pros1 is mainly secreted by hepatocytes where it acts as an anticoagulant, but Pros1 produced locally by other cells such as endothelial cells, osteoblasts, dendritic cells, T cells, vascular smooth muscle cells and tumour cells has no anticoagulant activity but instead functions as a cognate ligand for the TAM receptors Tyro3, Axl and Mertk. The two major functions of TAM receptors in adult tissues are the phagocytic uptake and clearance of apoptotic cells, and negative regulation of the immune system.^28^ Uncontrolled and prolonged inflammatory responses are essential contributing factors to the pathogenesis of chronic inflammation.^29^ TAM‐mediated clearance of apoptotic cells prevents the release of immunogenic cellular debris to the local environment, thereby limiting uncontrolled inflammatory response. The TAM receptors expressed in immune cells function to restrain innate immunity and inhibit the secretion of pro‐inflammatory cytokines.^30^, ^31^ In keeping with these functions of the TAM receptors to diminish inflammation, adult mice lacking all three TAM receptors were found to develop a severe lymphoproliferative disorder accompanied by broad‐spectrum autoimmunity, with hyperactivation of antigen‐presenting cells.^32^ As a cognate ligand for the TAM receptors, Pros1 participates in TAM‐mediated diminution of inflammation.^11^ Pros1 can simultaneously bind to Mertk expressed on the surface of a phagocyte and phosphatidylserine on an apoptotic cell, and consequently, activate Mertk‐mediated phagocytosis of the dying cell.^33^ In addition, Pros1 curbs inflammation by inhibiting TLR activation in dendritic cells^15^ as well as LPS‐stimulated expression of the pro‐inflammatory cytokines TNF‐, IL‐6 and IL‐1 by macrophages.^34^ Pros1 has also been reported to drive the growth and differentiation of NK precursors in vitro.^35^ Pros1 levels were found to be down‐regulated in several inflammatory diseases including SLE, inflammatory bowel disease (IBD) and renal vein and haemorrhagic rectocolitis,^36^, ^37^, ^38^ supporting the possible involvement of Pros1 in the pathogenesis of these diseases.

Prolonged overproduction of IFN‐I is implicated in the pathogenesis of periodontitis.^16^, ^17^, ^18^ Through association with IFNAR1, the TAM receptors can activate SOCS1/3 to inhibit IFN‐I production.^15^ The first evidence for the involvement of the TAM signalling in the development of periodontitis came from a recent study showing that the unrestrained IFN‐I production following *P gingivalis* infection was due to down‐regulation of TAM components.^19^ Specifically, repeated oral infections with *P gingivalis* led to MYD88 degradation and a reduced expression of Gas6, Axl and Pros1 regulated by MYD88. Although, MyD88 is generally essential for TLR2‐driven inflammation in response to *P gingivalis *or its isolated LPS while Pros1 also curbs inflammation by inhibiting TLR activation in dendritic cells,^15^ the significance of MyD88 may be different in biologically more relevant settings that include all cellular players. Indeed, *P gingivalis* manipulate MyD88 in immune cells and induce TLR2 signalling via alternative adaptors results in the induction of TLR2‐dependent, MyD88‐independent inflammation that leads to bone loss. The MyD88‐independent TLR2 activation induced by *P gingivalis* stimulates PI3K signalling that drives inflammation but at the same time depresses phagocytosis and enables phagocytosed bacteria to escape lysosomal degradation.^39^ Moreover, MyD88 promotes immune clearance of *P. gingivalis* 40 and the same pathogen induces MyD88 degradation in neutrophils,^41^ which is consistent with the findings in the oral tissue to suppress the bactericidal activity mediated by MyD88.^19^ Also, the resulting impairment in TAM signalling induced by MYD88 degradation reduced the expression of SOCS1/3, leading to uncontrolled IFN‐I expression by gingival epithelium. Consequently, T cells were constitutively activated and RANKL expression was raised, leading to alveolar bone resorption. In periodontitis patients, elevated Pros1 and decreased Tyro3 were detected in inflamed gingiva, while the expression of Gas6, Axl and Mertk was similar to that of healthy control.^19^

In this study, we also detected increased Pros1 and decreased Tyro3, but similar levels of Gas6, Axl and Mertk in inflamed human gingiva compared with healthy controls. Based on these data, we speculated that the Pros1/Tyro3 signalling may be the main TAM signalling involved in the development of periodontitis in humans. Indeed, results from our subsequent studies demonstrated that exogenous Pros1 inhibits *p.g*‐LPS–stimulated inflammation in cultured hGECs and ameliorates periodontal inflammation and alveolar bone loss in rats subjected to ligature and *p.g*‐LPS treatment. Similar to previous findings,^19^ Pros1 was mainly detected in the epithelium of human and murine gingiva. In previous cellular and animal studies, certain TAM components, and in particular, Pros1 were down‐regulated by LPS stimulation or repeated *P gingivalis* infection.^19^, ^34^ In this study, both Pros1 and Tyro3 were down‐regulated by *p.g*‐LPS stimulation, as observed in hGECs as well as in periodontitis rats. Of note, the down‐regulation of Pros1 in the in vitro and in vivo models of periodontitis was opposite to the up‐regulation of Pros1 in inflamed human gingiva of periodontitis patients. As periodontitis in humans is a chronic disease and can be caused by infection of a myriad of periodontal pathogens, the in vitro and in vivo models may not adequately represent the pathogenesis of periodontitis development in humans. The up‐regulation of Pros1 in chronically inflamed human gingiva may be developed as a mechanism to compensate for the loss of Tyro3 in order to control the inflammation. Pros1 has been shown to inhibit the expression of TNF‐α, IL‐6 and IL‐1β by macrophages in response to LPS stimulation in a TAM receptor‐dependent manner.^34^ In this work, Pros1 inhibited *p.g*‐LPs–induced production of TNF‐α, IL‐6, IL‐1β, MMP9/2 and RANKL by hGECs in a Tyro3‐dependent manner. In rats subjected to combinatorial treatment with ligature and *p.g*‐LPS, administration of Pros1 attenuated periodontitis‐mediated gingival inflammation, periodontal osteoclastogenesis and alveolar bone loss. Furthermore, our mechanistic studies implicated SOCS1/3 regulation of STAT1/3 as a mechanism of the in vitro and in vivo anti‐inflammatory effects of Pros1. Collectively, the findings from this work supported Pros1 as a novel therapy to combat periodontitis.

## CONFLICTS OF INTEREST

The authors confirm that there are no conflicts of interest.
