# Supplementary figures and images for: The Pros1/Tyro3 axis protects against periodontitis by modulating STAT/SOCS signalling
Source: J Cell Mol Med. 2019 Feb 7;23(4):2769–81. doi: 10.1111/jcmm.14183 (PMC6433735; doi:10.1111/jcmm.14183)

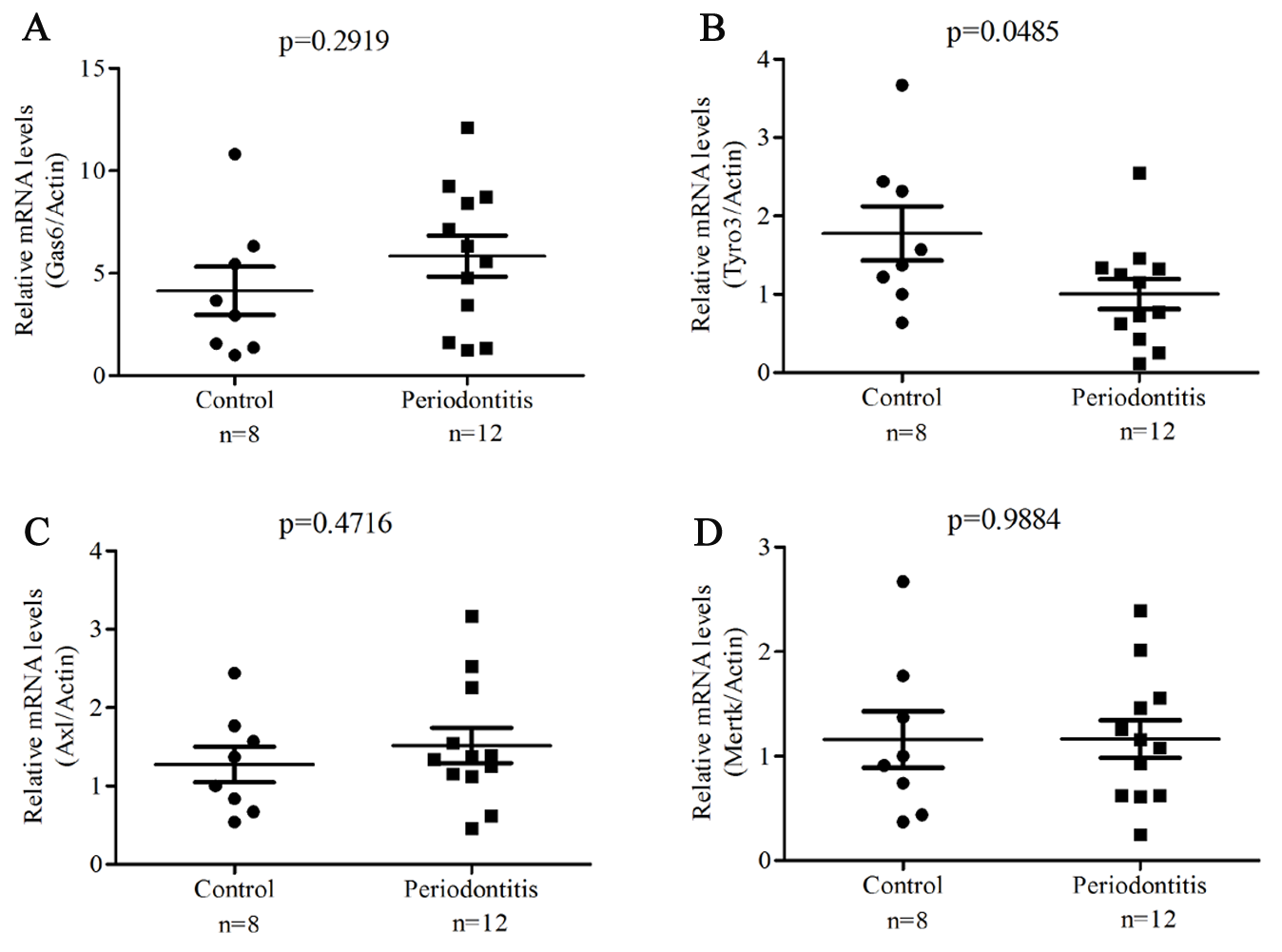

Supplement: Supplementary file 1 [file JCMM-23-2769-s001.tif]
